# Supplementary material for: Elucidating the potential carcinogenic molecular mechanisms of parabens in head and neck squamous cell carcinoma through network toxicology and molecular docking
Source: PLoS One. 2026 Mar 23;21(3):e0333867. doi: 10.1371/journal.pone.0333867 (PMC13008085; doi:10.1371/journal.pone.0333867)
Supplement: S1 Table — (DOCX) [file pone.0333867.s008.docx]

**Table S1 Toxicity prediction results of the selected parabens**

|  |  | Ethylparaben | Propylparaben | Methylparaben | Heptylparaben | Butylparaben | Benzylparaben |
| --- | --- | --- | --- | --- | --- | --- | --- |
| ProTox 3.0 | Molecular weight(g/mol | 166.17 | 180.2 | 152.15 | 236.31 | 194.23 | 228.24 |
|  | Predicted LD50(mg/kg) | 2500 | 3700 | 2000 | 1960 | 950 | 2500 |
|  | Predicted Toxicity Class* | 5 | 5 | 4 | 4 | 4 | 5 |
|  | Average similarity | 100% | 100% | 100% | 88.1% | 100% | 97.62% |
|  | Prediction accuracy | 100% | 100% | 100% | 70.97% | 100% | 72.9% |
| ADMETlab 2.0 | Bioconcentration Factors  -log10[(mg/L)/(1000*MW)] | 0.563 | 0.561 | 0.540 | 0.701 | 0.555 | 0.608 |
| ADMETlab 2.0 | IGC50  -log10[(mg/L)/(1000*MW)] | 3.663 | 3.906 | 3.434 | 4.996 | 4.279 | 4.492 |
|  | LC50FM  -log10[(mg/L)/(1000*MW)] | 3.915 | 4.001 | 3.401 | 5.111 | 4.274 | 4.764 |
|  | LC50DM  -log10[(mg/L)/(1000*MW)] | 4.669 | 4.757 | 4.235 | 4.949 | 4.826 | 4.908 |

*Class I: fatal if swallowed (LD50 ≤ 5); Class II: fatal if swallowed (5 < LD50 ≤ 50); Class III: toxic if swallowed (50 < LD50 ≤ 300); Class IV: harmful if swallowed (300 < LD50 ≤ 2000); Class V: may be harmful if swallowed (2000 < LD50 ≤ 5000); Class VI: non-toxic (LD50 > 5000). Bioconcentration Factors are used for considering secondary poisoning potential and assessing risks to human via the food chain. IGC50 means tetrahymena pyriformis 50 percent growth inhibition concentration. LC50FM means 96-hour fathead minnow 50 percent lethal concentration. LC50DM means 48-hour daphnia magna 50 percent lethal concentration.
